# Supplementary material for: Consequences of breed formation on patterns of genomic diversity and differentiation: the case of highly diverse peripheral Iberian cattle
Source: BMC Genomics. 2019 May 3;20:334. doi: 10.1186/s12864-019-5685-2 (PMC6500009; doi:10.1186/s12864-019-5685-2)
Supplement: Supplementary file 1 — Note S1. Brief description of the Iberian native cattle breeds sampled in our study. Table S1. Iberian breeds databases. Table S2. Individual sample information. The Y-chromosome haplogroups in bold were determined in this study (n.a.: not applicable). Table S3. FST values between taurine breeds. The highest value is shown in bold and the lowest in italic. Figure S1. Average error rate per sample. Figure S2. Population structure plots determined by NGSadmix; each individual is represented by a stacked column for 3, 4, 6 and 8 proportions. Other K values are shown in Fig. 1b. Figure S3. Principal component analysis done with PCAngsd (components 1 and 2 are shown in Fig. 1c). Variance explained by each component is shown in parenthesis. Figure S4. Differences in Tajima’s D between autosomes and sex chromosome X (calculated using only the female individuals). Figure S5. D-statistics determined as in [4] using genome-wide data. Positive values indicate an excess of derived alleles shared by the breeds in H2 (ANG: Angus; HOL: Holstein; JER: Jersey) and the British Aurochs [5], as indicated by the tree depicted above. Figure S6. Approximately-maximum-likelihood phylogeny of cattle Y-chromosome sequences (sites with a minimum of two minor alleles) determined in FastTree [6] which uses the Jukes-Cantor distance [7]. Labels for the Iberian cattle are according to Table S2. JER: Jersey; ANG: Angus; BOR: Borana; KEN: Kenana. 50% missing data was allowed. The taurine haplogroups Y1 and Y2 are shown in green and red, respectively, and the indicine Y3 in grey. Figure S7. FST per chromosome for all pairwise comparisons within Iberian cattle and between Iberian cattle and the African taurine N’Dama and the African indicine Ogaden. The proportion of shared variation oscillates throughout the genome, reaching extreme values for chromosome 21 (the largest between Iberian and African taurine cattle) and sex chromosome X (shows the lowest differentiation within taurine breeds, while hav [file 12864_2019_5685_MOESM1_ESM.docx]

Supplementary information

Supplementary Notes 2

Supplementary Tables 7

Supplementary Figures 13

References 20

## Supplementary notes

**Note S1. Brief description of the Iberian native cattle breeds sampled in our study**

The **Barrosã** cattle are one of the most emblematic of the Iberian Peninsula with their magnificent lyre-shaped horns and short face. These cattle can be found grazing in the highlands of northwestern Portugal in a collectively managed herding system named ‘vezeira’. They are medium-sized animals, with concave profile and brown-blond coat colour. There is marked sexual dimorphism and the males are much darker particularly in the neck and have a characteristic dark ring around the eyes. The herdbook was established in 1985 and is managed by the breeders’ association AMIBA (<http://www.amiba.pt>). The certified protected designation of origin (PDO) meat ‘Carne Barrosã’ is highly valued due to the intramuscular fat content and large numbers of live Barrosã cattle were exported to England from Oporto in the mid-19^th^ century until 1920. The milk of Barrosã cows has high fat content and was once used to produce butter. Barrosã cattle are used for draft and like Mirandesa they are also used in hierarchical bullfights (see below). There are about 9,000 and 331 breeding females and males, respectively, and this breed is thus considered not threatened by the Food and Agriculture Organization of the United Nations – FAO.

The **Maronesa** is a mountain breed well adapted to the rocky and steep terrain of ‘Serras do Alvão-Marão-Padrela’ in the interior north of Portugal. The calves are born a light fawn colour that turns to an almost black dark brown as they age, the adults may have a reddish stripe along the spine. Maronesa cattle have long horns that sweep forward, tilting down with points upward and outside. There is marked sexual dimorphism with the males being much heavier and taller than females. The herdbook was established in 1989 and is managed by the breeders’ association ACM (<http://www.marones.pt/>). They are used for draft and their certified meat known as ‘Carne Maronesa’ PDO has an excellent flavor. There are approximately 5,200 and 166 breeding females and males, respectively, with an average herd size of 5 animals. The Maronesa breed is considered by the FAO as being at risk of extinction.

The **Mirandesa** cattle were once the most abundant throughout the country with several regional varieties. Following a strong decrease in their population size, their production is mostly restricted to the northeastern region of Portugal in the Miranda plateau. They are large and compact animals that were developed for draft but are now mainly used for meat production. The Mirandesa cattle are classified within the brown concave group of Iberian breeds and they are known for their longevity. Their coat colour varies from light to dark brown in the extremities (males are darker), with a fringe of blond hair on the head and also along the spine. Their skin is pigmented in the nose and around the eyes. They have large horns that grow outward, bend down and then forward. The herdbook was among the first to be established in Portugal in 1959 and is managed by the breeders’ association ACBRM (<http://www.mirandesa.pt/>). The certified meat product ‘Carne Mirandesa’ PDO is widely recognized for its tenderness. The Mirandesa is also used in hierarchical bullfights known by ‘Chega de bois’, where the weaker animal usually backs away and the winner would often be selected for breeding as ‘boi do povo’. There are about 5,000 and ~230 breeding females and males, respectively, distributed by some 362 herds, and the breed is classified by the FAO as being at risk of extinction.

The **Arouquesa** breed is raised in a mountainous region south of the Douro river and other breeds from the north of Portugal are thought to have contributed to its formation, namely Barrosã, Mirandesa and Minhota. These cattle are small, with solid light brown (‘blond’) coat colour, dark nose and they have medium-sized curved horns with the dark points upward and outside. The herdbook was established in 1982 and is managed by the breeders’ association ANCRA (<http://www.ancra.pt/>). The Arouquesa animals feed mostly on natural resources, with an average of two cows per breeder used for draft, milk and meat. In older times their milk was highly valued due to the high fat content (~5.4%) to produce cheese and butter. Their meat is certified as ‘Carne Arouquesa’ PDO, and they also produce crossbred Mirandesa veal calves under the protected geographical indication ‘Vitela de Lafões’. There are less than 6,000 and ~170 breeding females and males, respectively, and the breed is classified by the FAO as being at risk of extinction.

The **Preta** breed can be found in the northeastern area of the Alentejo region and is known as ‘gado da terra’ or ‘charnequeiro’ because it feeds on very poor pastures and bush lands. These cattle are classified within the group of black Iberian breeds because of their solid black coat colour, black noses and hooves. They have wide lyre-shaped horns with dark points. The herdbook was established in 1993 and is managed by the breeders’ association ACBRP (<http://www.racapreta.com.pt/>). They are draught animals with high tolerance for hot climate, and their meat is certified as ‘Carne da Charneca’ PDO. There are less than 3,500 and 40 breeding females and males, respectively, distributed by some 30 herds and this breed is thus classified by the FAO as being at risk of extinction.

The **Brava de Lide** fighting cattle lives in semi-feral harsh conditions in the plains of ‘Ribatejo’. It is considered a hobby breed since it is mainly used in bullfights in association with the Lusitano horse. This breed is sub-divided in closed herds called ‘castas’ that are mainly owned by nobility and large estate ranchers. These cattle have a straight head profile characteristic of the Iberian black cattle and their coat colour is mainly black or dark brown, but it can vary from gray to white-patched, brindle, roan, red and chestnut. They have medium-sized horns, that spread out to the sides and are curved forward, with sharp upward points. They have a heavy dewlap and males are much larger than females. They are selected for aggressiveness and vigor, and they have long strong legs for agility. The herdbook was established in 1986 and is managed by the breeders’ association APBRB (<https://www.facebook.com/apbrb.lda>). Their meat is certified as ‘Carne de Bravo do Ribatejo’ PDO and has strong flavor and marbling characteristics. There are about 12,800 and 400 breeding females and males, respectively, distributed by some 100 herds, and Brava de Lide is classified as not threatened by the FAO.

The **Alentejana** cattle can be found in the Alentejo region in the south of Portugal (‘Transtagana cattle’, meaning from south of the Tagus river) and belong to the red convex group of Iberian breeds. These cattle have a solid red colour, long symmetrical horns and large body size. The males have strong and well-developed large necks, with an area of fat deposition named ‘cachaço’. The herdbook dates from 1968 and is currently managed by the breeders’ association ACBRA (<http://www.bovinoalentejano.pt/index.php?idm=1>). Alentejana cattle are well adapted to graze in extensive flatland pastures in a region of continental dry climate with large diurnal temperature range. They have a characteristic dewlap, typically with 7-folds of skin, for efficient sweating to cope with long periods of hot and dry summers. In older times they were used as draft animals but this breed is now specialized for meat production with certified PDO for its meat products known as ‘Carnalentejana’. There are approximately 25,000 and 270 breeding females and males, respectively, distributed by some 160 herds. This breed is the most numerous in Portugal and is thus classified as not threatened by the FAO (<http://www.fao.org/dad-is/browse-by-country-and-species/en/>).

The **Mertolenga** cattle are found in the southern Alentejo region and belong to the red convex group of Iberian breeds. They have a medium body size and there are three distinct coat colour varieties, in particular: solid red called ‘vermelho’; red roan known as ‘rosilho mil-flores’; and with red-brown spots on a white background named ‘malhado’. The herdbook was established in 1979 and is managed by the breeders’ association ACBM (<http://www.mertolenga.com/>). Mertolenga cattle are well adapted to graze the arid lowlands of Alentejo associated with areas of *Quercus suber* (cork tree) known as ‘montado’. They were once used as draught cattle but they are mainly raised for certified ‘Carne Mertolenga’ PDO beef production. The oxen known as ‘cabrestos’ are still used to herd fighting bulls and lead the bull out of the arena. There are about 15,285 breeding females 225 males, respectively, distributed by some 200 herds and the breed is thus classified as not threatened by the FAO.

References and additional information on Iberian native cattle:

- Ruralbit

<http://autoctones.ruralbit.com/index.php?esp=1&pais=pt>

- My Daily Cow® Portugal <http://krankykids.com/cows/mydailycow_alphabetical/P.html#portugal>
- Porter, V., L. Alderson, S. J.G. Hall, D. P. Sponenberg (2016). Mason's World Encyclopedia of Livestock Breeds and Breeding, 2 Volume Pack. Publisher CABI. ISBN 978 1 84593 466 8
- Domestic Animal Diversity Information System (DAD-IS), Food and Agriculture Organization of the United Nations – FAO

<http://www.fao.org/dad-is/browse-by-country-and-species/en/>

## Supplementary Tables

**Table S1.** Iberian breeds databases.

| Number of breeds | Country | Catalogue |
| --- | --- | --- |
| 15 | Portugal | http://www.dgv.min-agricultura.pt/xeov21/attachfileu.jsp?look_parentBoui=520984&att_display=n&att_download=y |
| 38 | Spain | http://www.mapama.gob.es/es/ganaderia/temas/zootecnia/razas-ganaderas/razas/catalogo/ |

**Table S2.** Individual sample information. The Y-chromosome haplogroups in bold were determined in this study (n.a.: not applicable).

| Breed | Sample | Mitochondrial haplogroup | Y-chromosome haplogroup | Sex (M=male; F=female) | Average depth in covered sites | Number of covered sites |
| --- | --- | --- | --- | --- | --- | --- |
| Alentejana | ALT08064 | T3 | H11Y2 | M | 2.1 | 121,603,199 |
| Alentejana | ALT08207 | Q | n.a. | F | 2.0 | 116,866,403 |
| Alentejana | ALT08210 | T1c1 | n.a. | F | 1.8 | 107,229,450 |
| Alentejana | ALT08796 | T3 | n.a. | F | 1.8 | 107,700,442 |
| Alentejana | ALT08798 | T123 | n.a. | F | 2.0 | 118,250,631 |
| Alentejana | ALT09280 | T1c1 | H11Y2 | M | 2.3 | 129,978,843 |
| Aroquesa | ARO09598 | T3 | H11Y2 | M | 1.8 | 109,658,921 |
| Aroquesa | ARO09612 | T3 | n.a. | F | 1.6 | 95,786,918 |
| Aroquesa | ARO09613 | T3 | H7Y2 | M | 2.1 | 120,146,159 |
| Aroquesa | ARO09620 | T1a | n.a. | F | 1.6 | 97,111,507 |
| Aroquesa | ARO09631 | T3 | n.a. | F | 1.7 | 96,955,139 |
| Aroquesa | ARO09686 | T3 | H6Y2 | M | 1.6 | 94,566,239 |
| Barrosã | BAR08911 | T3 | n.a. | F | 1.5 | 85,427,132 |
| Barrosã | BAR08916 | T1 | n.a. | F | 1.4 | 77,544,955 |
| Barrosã | BAR09270 | T3 | n.a. | F | 1.4 | 71,714,542 |
| Barrosã | BAR09274 | T3 | n.a. | F | 1.4 | 74,552,271 |
| Barrosã | BAR10099 | T3 | H6Y2 | M | 1.7 | 99,027,490 |
| Barrosã | BAR10145 | T1a | H12Y2 | M | 1.4 | 69,769,628 |
| Brava de Lide | BRA09567 | T3 | H3Y1 | M | 1.6 | 89,552,170 |
| Brava de Lide | BRA09569 | T3 | H5Y1 | M | 1.7 | 100,570,984 |
| Brava de Lide | BRA09640 | T3 | n.a. | F | 1.4 | 77,922,655 |
| Brava de Lide | BRA09825 | T1a | H6Y2 | M | 1.4 | 70,163,969 |
| Brava de Lide | BRA09826 | T1a | **Y2** | M | 1.7 | 105,491,751 |
| Brava de Lide | BRA10154 | T3 | n.a. | F | 1.5 | 86,386,748 |
| Maronesa | MRO09084 | T3 | H11Y2 | M | 1.7 | 100,912,249 |
| Maronesa | MRO09087 | T3 | n.a. | F | 1.8 | 109,812,848 |
| Maronesa | MRO09092 | T3 | H11Y2 | M | 1.6 | 95,958,843 |
| Maronesa | MRO09102 | T3 | n.a. | F | 1.5 | 80,751,938 |
| Maronesa | MRO09120 | T3 | **Y2** | M | 1.4 | 76,035,195 |
| Maronesa | MRO09123 | T3 | H11Y2 | M | 1.6 | 89,296,606 |
| Mertolenga | MER07804 | T3 | H10Y2 | M | 1.4 | 67,463,289 |
| Mertolenga | MER08113 | T3 | H11Y2 | M | 1.4 | 75,847,560 |
| Mertolenga | MER08116 | T2 | n.a. | F | 1.6 | 96,012,110 |
| Mertolenga | MER08190 | T3 | H6Y2 | M | 1.7 | 100,925,055 |
| Mertolenga | MER08769 | T123 | n.a. | F | 1.7 | 100,382,412 |
| Mertolenga | MER08776 | T3 | n.a. | F | 1.9 | 110,779,081 |
| Mirandesa | MIR08854 | T3 | n.a. | F | 1.4 | 74,057,988 |
| Mirandesa | MIR08859 | T3 | n.a. | F | 1.5 | 86,715,450 |
| Mirandesa | MIR08879 | T3 | H6Y2 | M | 1.7 | 103,560,835 |
| Mirandesa | MIR08880 | T1a | n.a. | F | 1.6 | 89,765,647 |
| Mirandesa | MIR08903 | T3 | H6Y2 | M | 1.7 | 103,100,599 |
| Mirandesa | MIR08906 | T3 | H6Y2 | M | 1.5 | 82,152,819 |
| Preta | PRE09295 | T1a | H8Y2 | M | 1.4 | 70,345,705 |
| Preta | PRE09409 | T3 | n.a. | F | 1.5 | 78,106,646 |
| Preta | PRE09555 | T3 | n.a. | F | 1.7 | 96,846,322 |
| Preta | PRE09947 | T3 | **Y2** | M | 1.6 | 88,651,129 |
| Preta | PRE09960 | T3 | n.a. | F | 1.3 | 61,641,160 |
| Preta | PRE09963 | T3 | H11Y2 | M | 1.5 | 80,291,901 |

**Table S3.** F_ST_ values between taurine breeds. The highest value is shown in bold and the lowest in italic.

|  | N'Dama | Jersey |
| --- | --- | --- |
| Holstein | 0.26 | 0.25 |
| N'Dama |  | **0.30** |

## Supplementary Figures


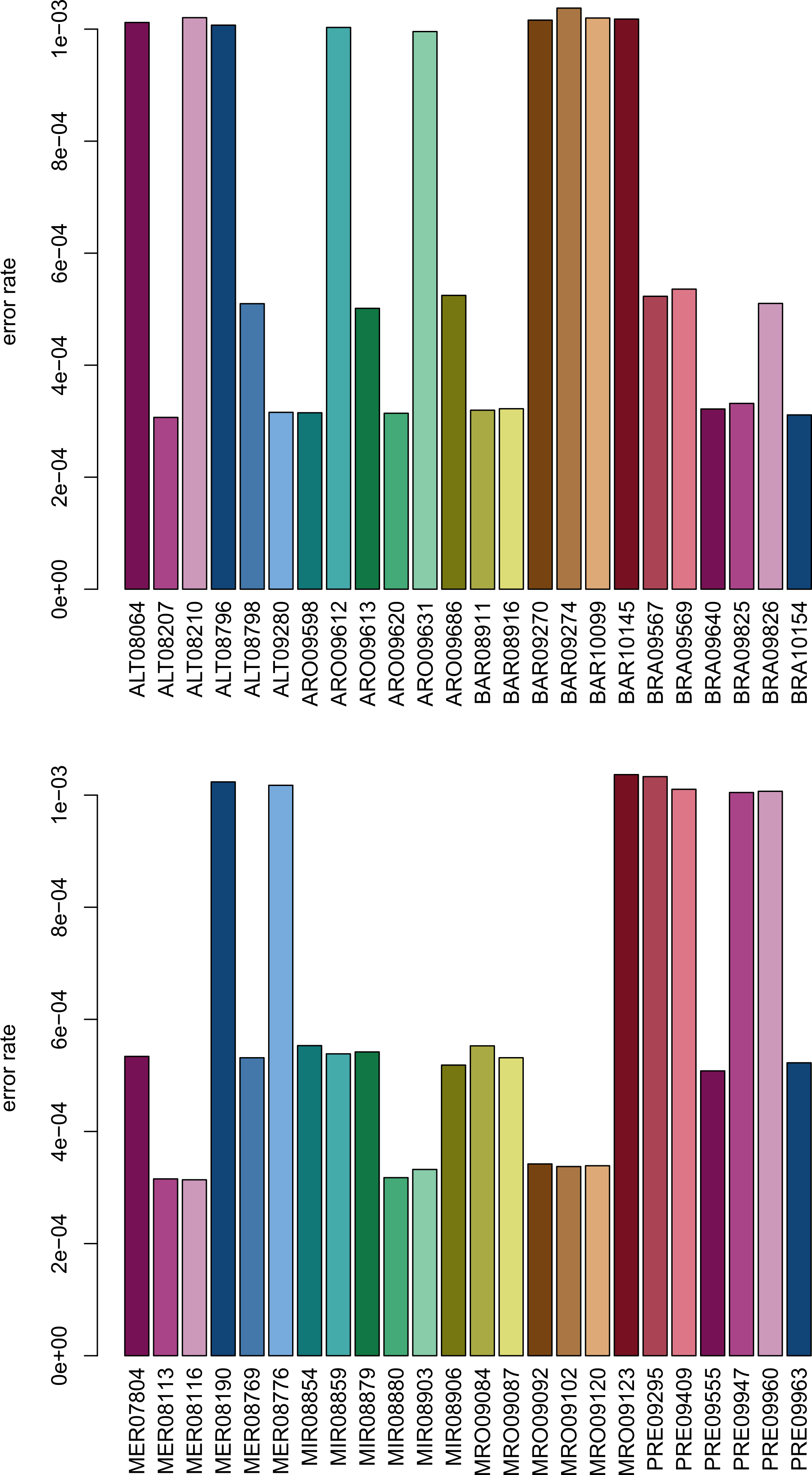


**Figure S1.** Average error rate per sample.


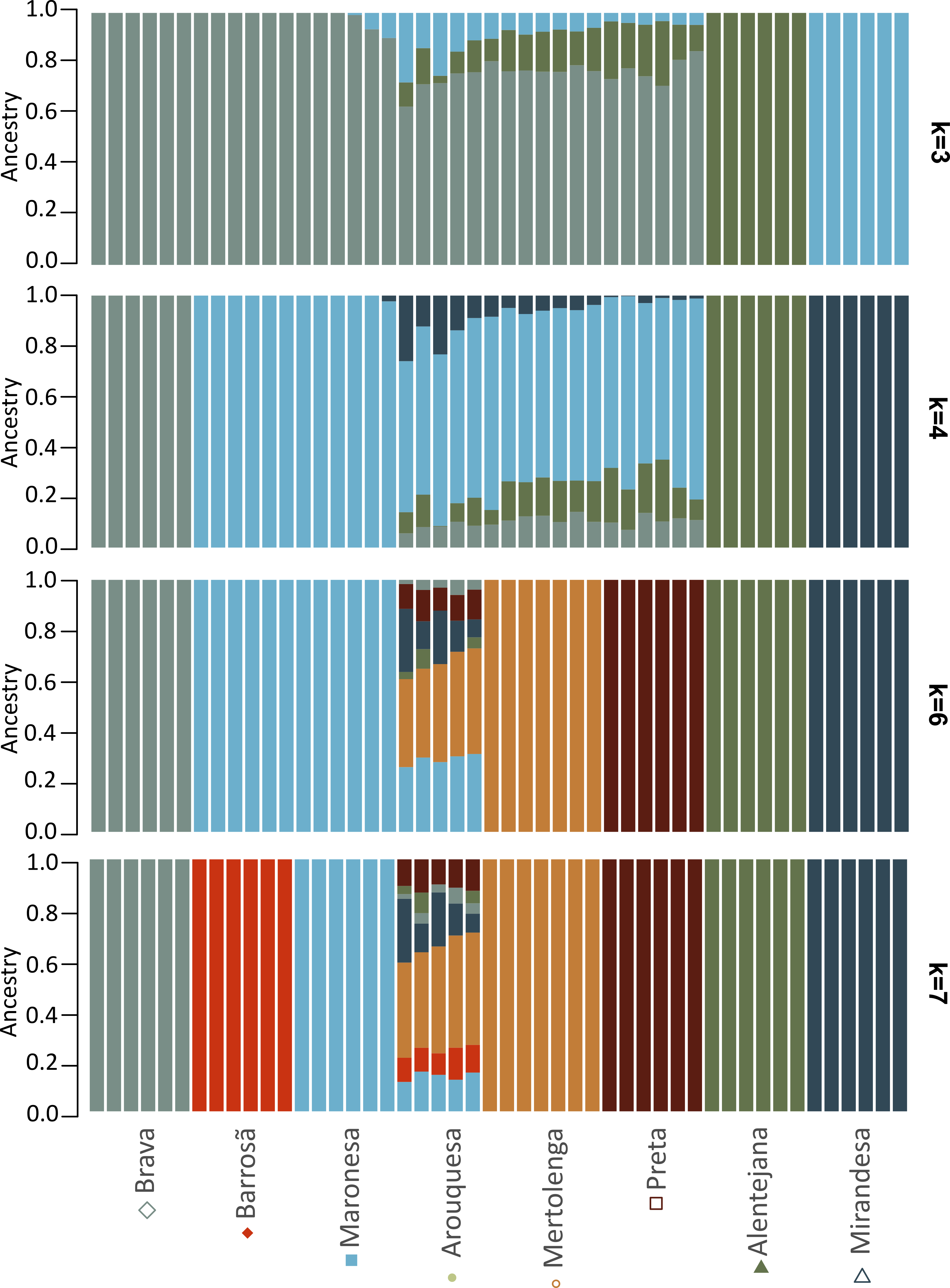


**Figure S2.** Population structure plots determined by NGSadmix; each individual is represented by a stacked column for 3, 4, 6 and 8 proportions. Other K values are shown in Figure 1.


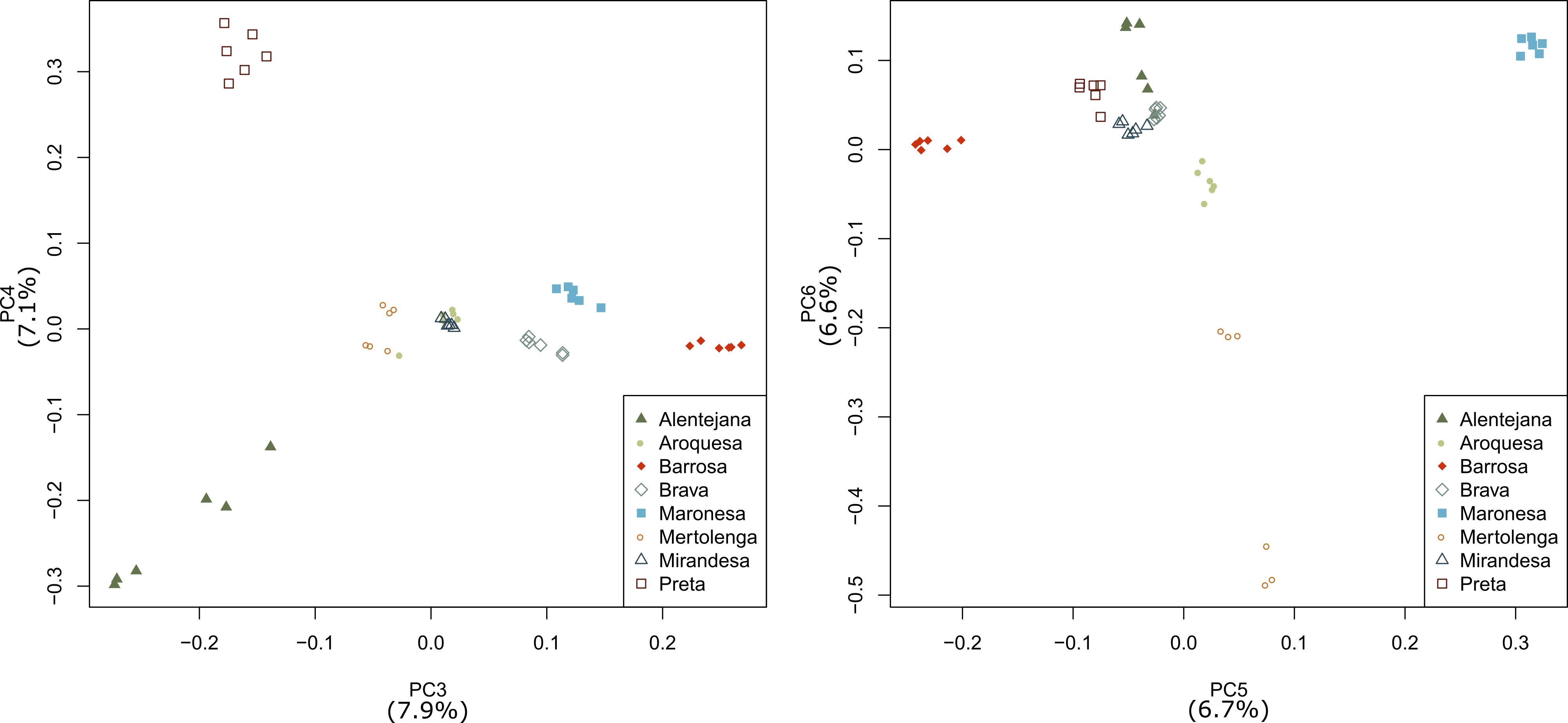


**Figure S3.** Principal component analysis done with PCAngsd (components 1 and 2 are shown in Figure 1). Variance explained by each component is shown in parenthesis.

**Figure S4.** Differences in Tajima’s D between autosomes and sex chromosome X (calculated using only the female individuals).


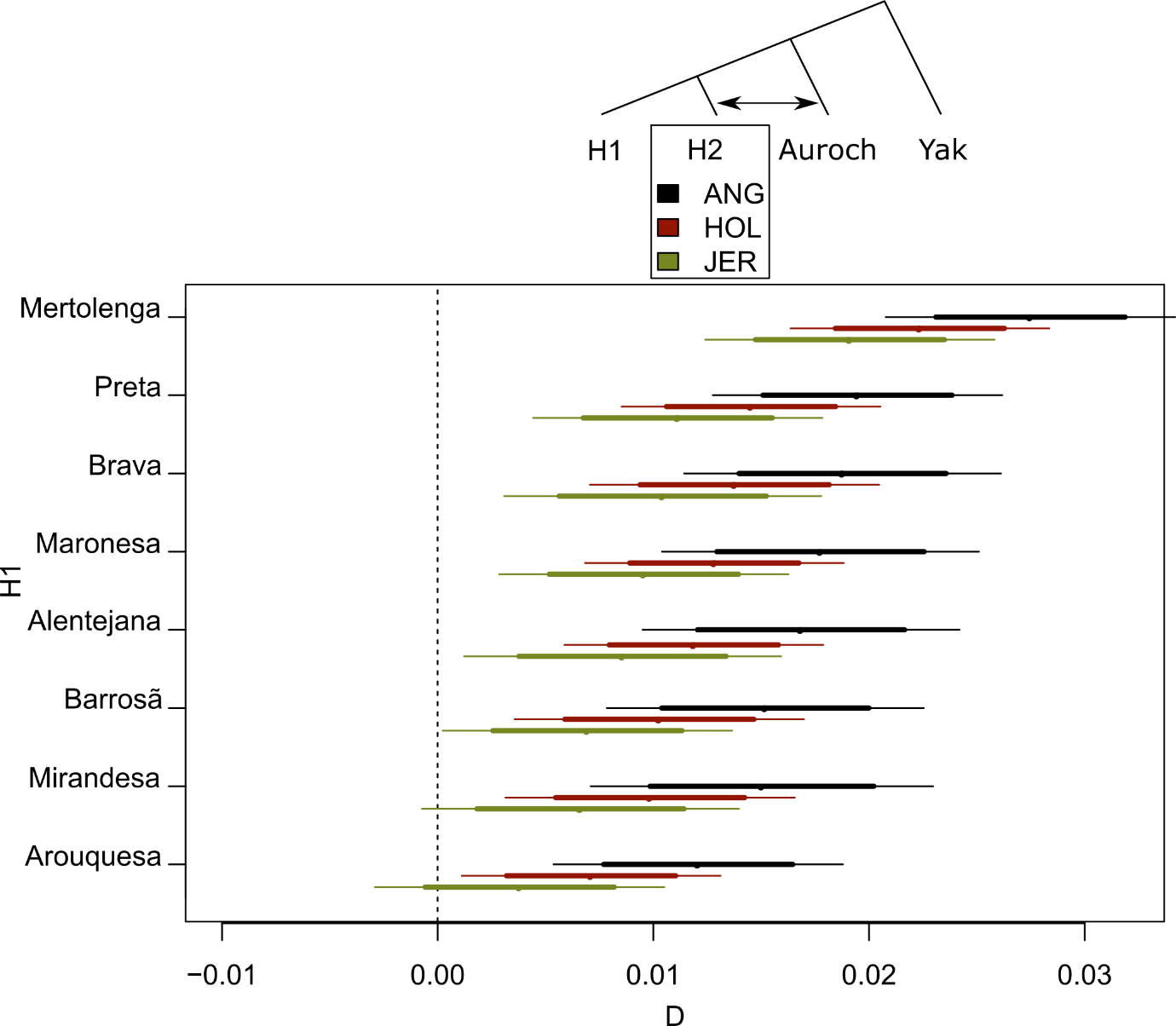


**Figure S5.** D-statistics determined as in [4] using genome-wide data. Positive values indicate an excess of derived alleles shared by the breeds in H2 (ANG: Angus; HOL: Holstein; JER: Jersey) and the British Aurochs [5], as indicated by the tree depicted above.


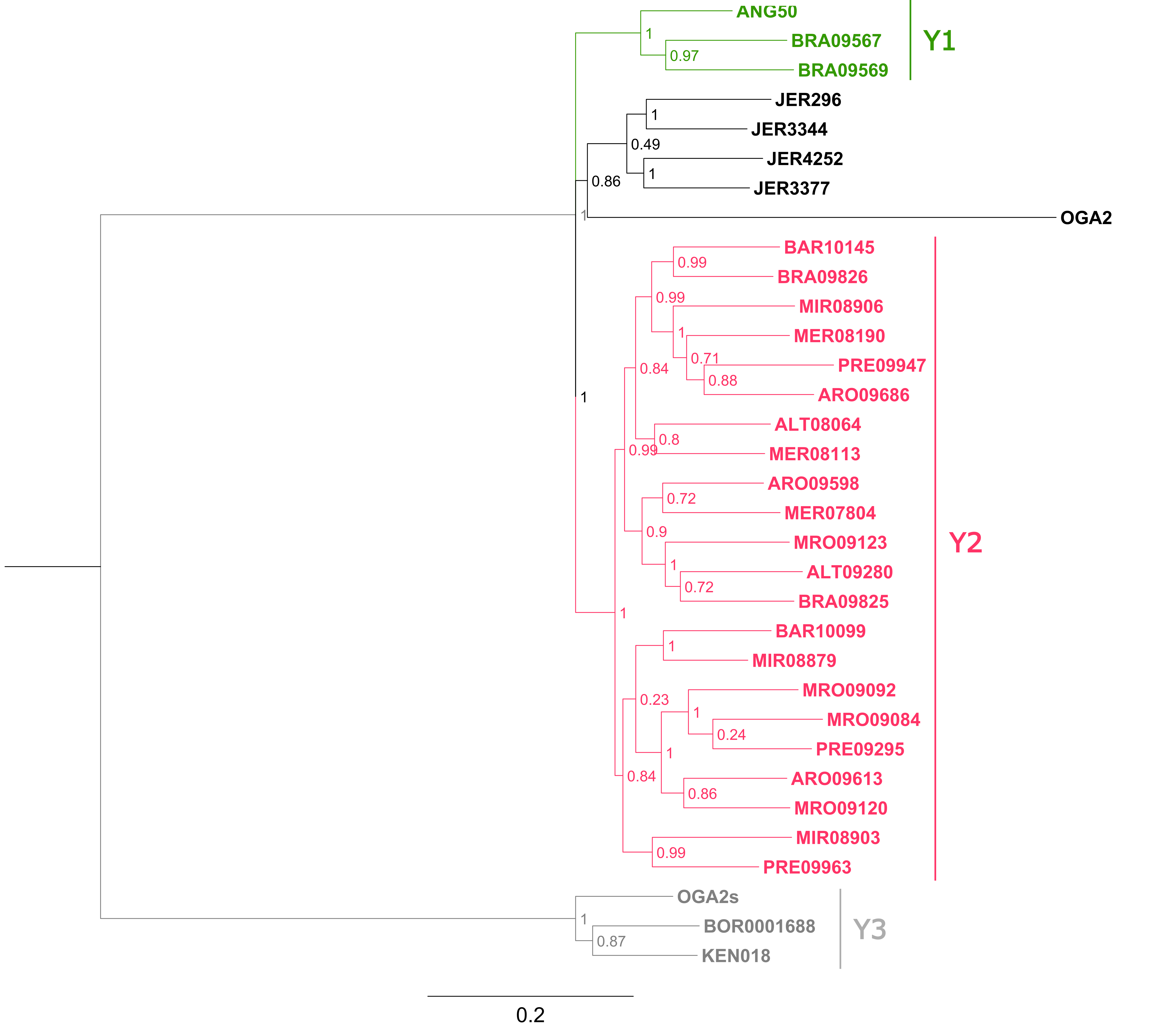


**Figure S6.** Approximately-maximum-likelihood phylogeny of cattle Y-chromosome sequences (sites with a minimum of two minor alleles) determined in FastTree [6] which uses the Jukes-Cantor distance [7]. Labels for the Iberian cattle are according to Table S2. JER: Jersey; ANG: Angus; BOR: Borana; KEN: Kenana. 50% missing data was allowed. The taurine haplogroups Y1 and Y2 are shown in green and red, respectively, and the indicine Y3 in grey.

**
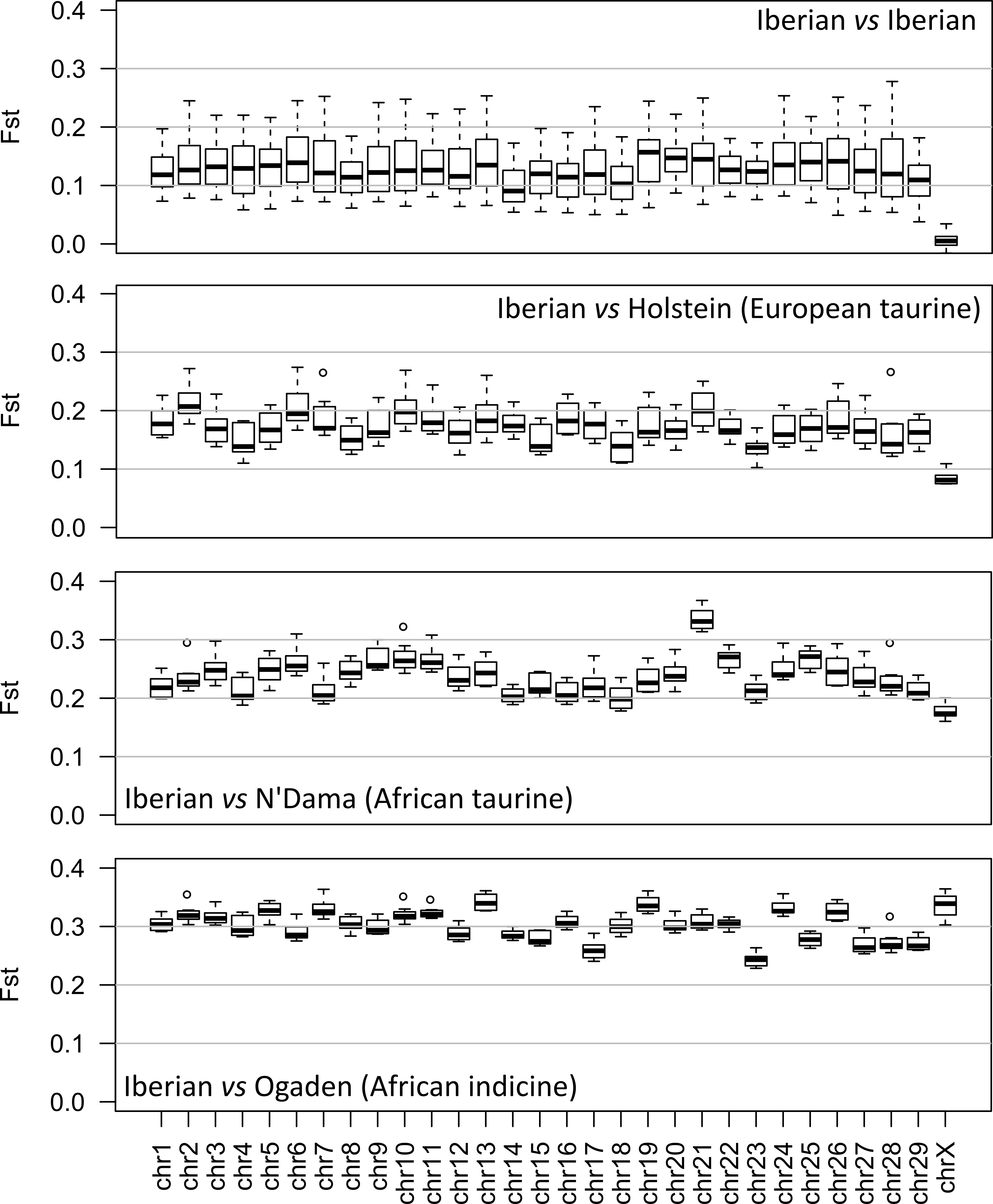
**

**Figure S7.** F_ST_ per chromosome for all pairwise comparisons within Iberian cattle and between Iberian cattle and the African taurine N’Dama and the African indicine Ogaden. The proportion of shared variation oscillates throughout the genome, reaching extreme values for chromosome 21 (the largest between Iberian and African taurine cattle) and sex chromosome X (shows the lowest differentiation within taurine breeds, while having a relatively large F_ST_ between Iberian and the African indicine cattle).

## References

1. Kim J, Hanotte O, Mwai OA, Dessie T, Bashir S, Diallo B, et al. The genome landscape of indigenous African cattle. Genome Biol. [Internet]. 2017 [cited 2018 Apr 26];18:34. Available from: https://genomebiology.biomedcentral.com/track/pdf/10.1186/s13059-017-1153-y

2. Upadhyay MR, Chen W, Lenstra JA, Goderie CRJ, MacHugh DE, Park SDE, et al. Genetic origin, admixture and population history of aurochs (Bos primigenius) and primitive European cattle. Heredity (Edinb). [Internet]. Nature Publishing Group; 2017 [cited 2018 Apr 26];118:169–76. Available from: http://www.nature.com/articles/hdy201679

3. Qiu Q, Zhang G, Ma T, Qian W, Wang J, Ye Z, et al. The yak genome and adaptation to life at high altitude. Nat. Genet. [Internet]. 2012 [cited 2018 Jul 3];44:946–9. Available from: http://www.ncbi.nlm.nih.gov/pubmed/22751099

4. Soraggi S, Wiuf C, Albrechtsen A. Powerful Inference with the D-Statistic on Low-Coverage Whole-Genome Data. G3 (Bethesda). [Internet]. G3: Genes, Genomes, Genetics; 2018 [cited 2018 Apr 17];8:551–66. Available from: http://www.ncbi.nlm.nih.gov/pubmed/29196497

5. Park SDE, Magee DA, McGettigan PA, Teasdale MD, Edwards CJ, Lohan AJ, et al. Genome sequencing of the extinct Eurasian wild aurochs, Bos primigenius, illuminates the phylogeography and evolution of cattle. Genome Biol. [Internet]. BioMed Central; 2015 [cited 2018 Apr 30];16:234. Available from: http://genomebiology.com/2015/16/1/234

6. Price MN, Dehal PS, Arkin AP. FastTree: computing large minimum evolution trees with profiles instead of a distance matrix. Mol. Biol. Evol. [Internet]. 2009 [cited 2014 Nov 5];26:1641–50. Available from: http://mbe.oxfordjournals.org/content/26/7/1641.full

7. Jukes TH, Cantor CR. Evolution of protein molecules. In: HN M, editor. Mamm. Protein Metab. New York, USA: Academic Press; 1969. p. 21–132.
